# Addressing Depression Comorbid With Diabetes or Hypertension in Resource-Poor Settings: A Qualitative Study About User Perception of a Nurse-Supported Smartphone App in Peru

**DOI:** 10.2196/11701

**Published:** 2019-06-18

**Authors:** Lena R Brandt, Liliana Hidalgo, Francisco Diez-Canseco, Ricardo Araya, David C Mohr, Paulo R Menezes, J Jaime Miranda

**Affiliations:** 1 CRONICAS Center of Excellence in Chronic Diseases Universidad Peruana Cayetano Heredia Lima Peru; 2 Centre for Global Mental Health and Primary Care Research, Health Service and Population Research Institute of Psychiatry, Psychology and Neuroscience King’s College London London United Kingdom; 3 Center for Behavioral Intervention Technologies Northwestern University Chicago, IL United States; 4 Faculdade de Medicina Universidade de São Paulo São Paulo Brazil; 5 Population Mental Health Research Centre Universidade de São Paulo São Paulo Brazil; 6 School of Medicine Department of Medicine Universidad Peruana Cayetano Heredia Lima Peru

**Keywords:** mental health, depression, noncommunicable diseases, mHealth, smartphone, developing countries

## Abstract

**Background:**

Smartphone apps could constitute a cost-effective strategy to overcome health care system access barriers to mental health services for people in low- and middle-income countries.

**Objective:**

The aim of this paper was to explore the patients’ perspectives of CONEMO (*Emotional Control*, in Spanish: *Control Emocional*), a technology-driven, psychoeducational, and nurse-supported intervention delivered via a smartphone app aimed at reducing depressive symptoms in people with diabetes, hypertension or both who attend public health care centers, as well as the nurses’ feedback about their role and its feasibility to be scaled up.

**Methods:**

This study combines data from 2 pilot studies performed in Lima, Peru, between 2015 and 2016, to test the feasibility of CONEMO. Interviews were conducted with 29 patients with diabetes, hypertension or both with comorbid depressive symptoms who used CONEMO and 6 staff nurses who accompanied the intervention. Using a content analysis approach, interview notes from patient interviews were transferred to a digital format, coded, and categorized into 6 main domains: the perceived health benefit, usability, adherence, user satisfaction with the app, nurse’s support, and suggestions to improve the intervention. Interviews with nurses were analyzed by the same approach and categorized into 4 domains: general feedback, evaluation of training, evaluation of study activities, and feasibility of implementing this intervention within the existing structures of health system.

**Results:**

Patients perceived improvement in their emotional health because of CONEMO, whereas some also reported better physical health. Many encountered some difficulties with using CONEMO, but resolved them with time and practice. However, the interactive elements of the app, such as short message service, android notifications, and pop-up messages were mostly perceived as challenging. Satisfaction with CONEMO was high, as was the self-reported adherence. Overall, patients evaluated the nurse accompaniment positively, but they suggested improvements in the technological training and an increase in the amount of contact. Nurses reported some difficulties in completing their tasks and explained that the CONEMO intervention activities competed with their everyday work routine.

**Conclusions:**

Using a nurse-supported smartphone app to reduce depressive symptoms among people with chronic diseases is possible and mostly perceived beneficial by the patients, but it requires context-specific adaptations regarding the implementation of a task shifting approach within the public health care system. These results provide valuable information about user feedback for those building mobile health interventions for depression.

## Introduction

Mental health disorders are one of the main health burdens worldwide. Of the 20 leading causes of years lived with disability in the world, 9 of them are mental, neurological or substance use disorders [1]; neuropsychiatric disorders make a contribution of 13% to 14% to the global burden of disease, measured by the disability-adjusted life years (DALYs), because of the mortality and years of life lost derived from the time lived with compromised health [2-4]. In Peru, neuropsychiatric disorders are even considered as the ones that cause the highest burden of disease in the country, with a contribution of 16% to the DALYs [5]. Furthermore, unipolar depression is the main cause of burden of disease among this group [3] and is even higher among those with comorbid chronic noncommunicable diseases (NCDs), such as diabetes or hypertension [6-12]. Considering this high comorbidity, the great incidence of NCDs in low- and middle-income countries (LMICs) [13,14], as well as the important possible negative consequences of this relationship on health outcomes [6,11,12,14-18] and adherence to treatment [8,16], interventions specifically designed for individuals with depression and comorbid diabetes, hypertension or both are needed.

Access to mental health services is poor across LMICs. For example, in Peru, most health insurance does not cover mental health care [5]. Furthermore, there are far fewer psychologists and psychiatrists per inhabitant than in other upper- and middle-income countries, and of those who are available, 85% are located in Lima, the Peruvian capital [19]. Only 24.3% of the population in Lima with mental health disorders receive any mental health care [20], which is even less than 10.4% in rural Andean regions [21]. One approach to confront system-related barriers for mental health care is task shifting [22-24]. Shifting tasks within health care from a specialized professional to a person not specialized in the same field, for example, from a psychiatrist to a comprehensively trained nurse or other health worker not specialized in mental health, is a cost-effective [25,26] and cost-saving approach [27-31]. Similarly, it has shown promising results for a wide variety of health outcomes, including mental health conditions [32,33] such as depression [29,33], and could therefore be an ideal option for LMIC settings to amplify the access to health care [34] where human resources are scarce.

Other strategies to overcome health system barriers and make mental health care more accessible to the community [35,36] are mobile health (mHealth) [35,37-41] and other digital self-help interventions [42-45], which have been shown to improve mental health–related outcomes. There are several mobile depression apps already on the market, but in addition to stand-alone, unguided self-management apps, linking interventions to existing resources from local health care systems merits exploration. Furthermore, many of the *depression apps* currently on the market often lack a solid theoretical background or do not follow clinical guidelines that have already proven to be effective [46,47], and besides, most of them are not available in Spanish. Evidence from high-income countries indicates that the effectiveness of and adherence to digital interventions is higher when the health app is easy to use [39,48,49], is individually tailored [46], and includes human interaction [48,50-52]. Hence, it is crucial to evaluate the users’ perspective and feasibility of using mHealth apps in the LMIC context. Although they are not ubiquitous yet, as pervasiveness of smartphones is continuously increasing in developing countries [53,54], mHealth apps will become more needed in the near future. In Peru, the access to smartphones increased from 14.8% to 30.8% among people aged between 46 and 50 years and from 6.8% to 18.0% in those aged 51 years or older in only 1 year (2014-2015) [55], and it is expected that these percentages will continue to grow.

The Latin America Treatment and Innovation Network in Mental Health (LATIN-MH) [56] integrated these strategies and developed a new smartphone app, CONEMO (*Emotional Control*, in Spanish: *Control Emocional*), a technology-driven, psychoeducational, and nurse-supported intervention aimed at reducing depressive symptoms in people with diabetes, hypertension or both. This study is derived from the qualitative formative research conducted in Lima, Peru, in preparation for a randomized controlled trial testing the effectiveness of CONEMO. The aims of this study were to explore (1) the experience of patients using CONEMO with regard to perceived health benefit, usability, adherence to CONEMO, satisfaction, suggestions for CONEMO, as well as the evaluation of the nurse-support received and (2) the experience of nurses supporting CONEMO users regarding general nurse feedback, evaluation of training received, evaluation of activities related to the study, and their perception of the feasibility of using this type of *self-management-plus-coaching* approach within the Peruvian public health care system. The quantitative outcomes of the pilot studies will be reported elsewhere [57].

## Methods

### Study Design and Theoretical Framework

This study analyzes the qualitative information acquired from interviews with patients and nurses about their experiences in 2 pilot studies. Interviews were selected as the most suitable technique, which were applied after their participation in these studies. The data were analyzed using qualitative content analysis, as, for example, described by Bengtsson [58], considering it as “a research method that provides a systematic and objective means to make valid inferences from verbal, visual, or written data in order to describe and quantify specific phenomena” [59].

### Setting

The 2 pilot studies were conducted between 2015 and 2016 in Lima; the first pilot study was implemented in 1 general hospital of the Ministry of Health (MINSA) and the second in 2 primary health care centers of EsSalud, Peru’s Social Security System. Those are 2 distinct public health care systems in Peru, which differ in their organization and functioning. The reason for conducting 2 pilot studies was, first, to pilot test the implementation in the 2 different national health care systems, and second, to test the feasibility of working with staff nurses from the health care system within this study instead of hired nurses, which was not possible in the first pilot study in the MINSA hospital.

### Participant Selection

#### Sampling

The study integrated data from 2 groups of participants: the patient’s experience with CONEMO and the nurses’ experience of conducting the nurse-support of CONEMO within the health care system.

Using a convenience sampling strategy, patients were selected based on the following criteria: having a diagnosis of diabetes, hypertension or both by a physician or receiving treatment for it, being aged at least 21 years, being able to read and write in Spanish (all of the above were self-reported), and experiencing clinically significant depressive symptoms, as measured by the Patient Health Questionnaire-9 (cutoff score ≥10) [60]. Patients who were pregnant or showed cognitive impairment (measured with the Brief Community Screening Instrument for Dementia [61]) or psychotic symptoms (measured with the Psychosis Screening Questionnaire [62]) were excluded.

In the first pilot study, implemented in a hospital of the MINSA, 1 nurse was hired by the project. In the second pilot study, nurses from the Social Security System were selected and assigned to the study by the health care centers’ administration. In 1 health care center, all 5 nurses who were working there at the time were assigned to participate in the study, whereas in the other health care center, 1 nurse who was working in the elderly adult program was selected.

#### Method of Approach

In the MINSA hospital, recruitment took place in the endocrinology and cardiology outpatient clinics, whereas in the 2 primary health care centers, patients were recruited in the waiting areas or in the elderly adult consultation unit that monitors people with NCDs. Some patients were approached before or after their regular consultations, whereas others were referred to the fieldwork team by health care providers. Patients were screened by 1 of 4 fieldworkers who were all psychologists. If the inclusion criteria were fulfilled, they were invited to participate in the 6-week study and to sign a consent form.

Afterward, patients were invited to return to the health care center to complete a baseline questionnaire. Subsequently, they were assigned to a nurse who then scheduled an individual appointment with each patient to train them and provide the study materials. As, at the time of the pilot, smartphones were not yet ubiquitous among low-income Peruvians, all patients were given an Android smartphone with CONEMO installed as well as 2 guidebooks: one about how to use the smartphone and CONEMO and one about the research project. The nurses trained the patients in the use of the technology and informed them that their activities could be followed on a Web-based platform and that they would receive at least 2 monitoring phone calls throughout the intervention period to address difficulties if applicable.

The nurses were approached via the centers’ administration. After the managers of the centers accepted to participate, they selected the nurses and facilitated the contact to the research team. Afterward, all nurses underwent a 3-month theoretical and practical training course with 1 to 2 hours of weekly training sessions to perform their activities before starting to receive patients.

#### Sample Size and Nonparticipation

Over the 2 pilot studies, 45 patients signed the consent form to participate, but 12 of them did not return to the health care center to receive CONEMO. Of the 33 patients who finally received CONEMO, 29 responded to the interviews (15 of pilot study 1, monitored by a hired nurse, and 14 of pilot study 2, monitored by staff nurses from the health care system).

With regard to the nurses, 6 staff nurses, who were working within the Social Security System and who participated in the second pilot study, were interviewed after finalizing the pilot study activities. The nurse hired in the first pilot study was not considered in the interviews, considering that she would not represent the perspectives of someone working within the health care system.

### Intervention: CONEMO (Emotional Control)

CONEMO is a technology-driven, psychoeducational, and nurse-supported intervention that was delivered via a smartphone app (see Multimedia Appendix 1 for a screenshot of CONEMO). A literature review was conducted to develop both the content, which was based on the theoretical framework of behavioral activation [63-66], as well as the design and structure of the CONEMO app.

Behavioral activation aims at reducing depression by motivating patients to identify and complete activities to obtain a sense of pleasure and accomplishment [66]. In that sense, the content of the intervention included psychoeducation about depression, various lists of potential activities that patients could be interested in, motivational content to stimulate activity completion, as well as strategies to help patients complete these activities. These content elements were delivered in the form of both text and videos. The videos featured a middle-aged woman representing a health practitioner who did not embody any specific social class or background.

Providing a list of suggested activities to the participant was also used in other apps based on behavioral activation to lower depressive symptoms [67,68]. Previous behavioral activation research suggested a list of 11 activity categories in which patients could start doing activities [66]. Owing to the length of our intervention, those 11 domains were regrouped into 3 categories: pleasant activities (eg, based on hobbies and entertainment among others, such as reading a book or meeting friends), healthy activities (based on sports and health, such as following their indicated diet and exercising), and tasks (based on domestic activities, which included activities such as cleaning the house). In this process, activities were discussed and adapted by the research team to ensure the appropriateness of the listed activities, considering the target population’s health characteristics and culture. Finally, CONEMO included interactive elements, such as Android notifications and dialogue pop-ups to remind patients to complete their sessions and activities and let them give feedback, short message service (SMS) text messaging to remind them of their appointments with the nurse, as well as an option to request help from the nurse.

For the development of the structure of the CONEMO app, other behavioral activation intervention outlines, as well as existing mobile intervention designs, were considered. Although in-person interventions based on behavioral activation usually have a duration of 5 to 12 weekly sessions, with each session lasting approximately 45 min [69,70], interventions using technology need to deliver the core elements of the treatment by adapting them to the interface used and not as a mere copy of the content [71]. Other mobile interventions using behavioral activation were typically designed for a duration of 8 to 10 weekly sessions but were also accompanied with weekly psychotherapy sessions [67,68] or pharmacotherapy [72]. Considering the nurse accompanying the CONEMO intervention would only give technical support and that app developers recommend designing interventions of “high frequency, low intensity, and shorter time commitment” [71], it was decided to include 18 sessions in total, delivered 3 times a week over a period of 6 weeks. The completion time for each session (excluding the suggested outside-app activity) was estimated to take between 5 and 10 min.

The role of the nurses was to train all patients in the use of the app and the smartphone, followed by regular monitoring of the patients’ participation throughout the 6 weeks of intervention via a Web-based platform, the *nurse dashboard*. The nurse-support protocol was based on the principles of supportive accountability [52], with the aim of maintaining patient engagement. Nurses were instructed to contact the patient periodically to positively reinforce use, inquire if they had any difficulties, and resolve possible problems with using CONEMO. In addition, the nurse dashboard served as an activity and progress record, allowing nurses to log their activities, such as the completed phone calls or other contacts they had with the patients. The roughly estimated time expected to be invested per patient was around 2.5 hours in total over the 6-week period (45 to 60 min for the first appointment used for training, 15 to 30 min for the last appointment, and 15 min per remaining week for follow-up calls). Furthermore, during the intervention, all nurses took part in weekly supervision meetings with a psychologist, in which particular cases, tasks, and difficulties were addressed. These supervision meetings were expected to take around 15 to 45 min weekly.

The technology and intervention used in this study was created in collaboration with representatives from the Universidad Peruana Cayetano Heredia in Lima, Peru, and the University of São Paulo in São Paulo, Brazil. Northwestern University’s Center for Behavioral Intervention Technologies in Chicago, United States, supported the design work and provided all software programming. CONEMO was developed to be adaptable linguistically and culturally, providing versions in Spanish, Portuguese, and English.

### Data Collection

After the 6 weeks of intervention, the trial manager (LRB) and the clinical coordinator (LH)—both female psychologists—conducted the interviews with the patients and nurses. During both the patient and nurse interviews, the interviewers aimed to transcribe as closely as possible the literal content of the participants’ responses and later transferred these notes to the computer to reduce information loss. During some of the patient interviews and all of the nurse interviews, both interviewers took notes simultaneously, compared them afterward, and found high literal consistency between the notes. Subsequently, the digitized notes were integrated into 1 document.

The patients’ semistructured interviews were applied, consisting of 30 questions addressing the 6 areas of interest, which were considered as most important indicators in view of the randomized controlled trial to be implemented subsequently: perceived health benefit, usability, adherence to CONEMO, satisfaction and suggestions for CONEMO, as well as the evaluation of the nurse-support received (see Multimedia Appendix 2 for the interview guide for patients). Satisfaction refers here to the patients’ evaluation of things they liked and disliked about CONEMO. Each interview took between 45 and 60 min.

With the nurses, semistructured interviews consisting of 22 questions to retrieve data about general nurse feedback, evaluation of training received, evaluation of activities related to the study, as well as their perception of feasibility of incorporating CONEMO within primary care (see Multimedia Appendix 3 for the interview guide for nurses) were conducted. Feasibility refers here to the nurses’ perceptions of the viability to scale up the nurse-supported CONEMO within similar health care centers and to be implemented by staff nurses. Each interview took around 40 min. All interviews were conducted in 1 session, without conducting repeat interviews.

### Data Analysis

The 4 stages of content analysis— *decontextualization, recontextualization, categorization*, and *compilation* —as described by Bengtsson [58], were conducted by 2 researchers—the ones who also conducted the interviews—to increase validity and decrease bias, as suggested by various authors [58,73,74]. First, the digitized interview notes for patients, as well as for nurses, were reviewed, and thereby, codes were generated (*decontextualization*). Both researchers discussed their comprehension of each code and adapted them accordingly before actually coding the data. Then, they compared the codes with the original data to control for completeness of the codes (*recontextualization*). Codes were created for the complete dataset, so that every statement made by a person would be assigned to at least 1 code. The aim was to have at least 1 code for every statement, which would not overlap in its meaning with other codes. Therefore, initially, no information was considered *dross* [58,73]. After the codification process, the researchers revised the codification of the other person and discussed and adapted discrepancies to control for different interpretations and increase compatibility.

On the basis of this principle, the qualitative data from patients were first divided into 14 principal codes and 39 subcodes (see Multimedia Appendix 4 for complete patient codebook). After a thorough review, data from 11 principal codes and 27 subcodes were assembled to the 6 domains of interest (*categorization*). The data from the remaining codes were considered as not relevant to the research aim and therefore as *dross* (*recontextualization*). Table 1 provides a description of the principal codes and subcodes used to describe each domain. The qualitative data from the nurses were coded into 9 principal codes and 18 subcodes (see Multimedia Appendix 5 for complete nurse codebook), of which data from 16 subcodes over all of the 9 principal codes were selected for this analysis and subsequently categorized into the 4 domains of interest, which are described in Table 2.

Afterward, the data were analyzed within each of the domains (*compilation*). A manifest analysis technique [58,59,74] was chosen to describe the experience narrated by the informants. Although our analysis was based on qualitative content analysis, in order to draw conclusions from the information retrieved, elements of quantitative content analysis were also applied to receive information about how common certain perspectives were within this population, as suggested by other authors [58,75,76].

### Ethical Considerations

The formative research and its materials were approved by the Institutional Ethics Committee of the Universidad Peruana Cayetano Heredia in Lima, Peru, as well as by the Data and Safety Monitoring Board of the National Institute of Mental Health in the United States, in accordance with applicable regulations. Informed consent was obtained from all the participants before participating in the study, after screening for fulfillment of inclusion criteria, and having conducted a thorough explanation of the study and all implications of participation. Patients were not paid, but they were reimbursed for transportation costs related to this study.

**Table 1:** Domains and codes used for patients’ interviews’ analysis.

Domains	Principal codes	Subcodes
Perceived health benefit of CONEMO	Perceived health benefit of CONEMO	Perceived benefit on psychological health, perceived benefit on physical health, and other benefits perceived
Usability	Usability of CONEMO	Difficulties with CONEMO usage, and help from others
	Evaluation of smartphone	Difficulties with smartphone usage
	Guidebooks	Revision of guidebooks, and utility of guidebooks
	Interactive tools (notifications, dialogue pop-ups and SMS)	—^a^
Adherence to CONEMO	Adherence to CONEMO	Revision of sessions, performing activities, and difficulties (to perform activities)
Satisfaction with CONEMO	Satisfaction with CONEMO	General feedback, things most liked about CONEMO, things least liked about CONEMO, things most liked about the sessions, things least liked about the sessions, and using smartphones for intervention delivery
Suggestions for CONEMO	Design	Design of CONEMO
	Duration and frequency	Duration of intervention, and frequency of sessions
	Suggestions for CONEMO	Suggestions to improve CONEMO, and preference of how to receive information
Evaluation of nurse component	Evaluation of nurse component	Evaluation of training, quantity of contacts, evaluation of contacts, help requests, and suggestions for the nurse component

^a^For some principal codes, further subdivision did not seem to be beneficial; therefore, no subcodes were created.

**Table 2:** Domains and codes used for nurses’ interviews’ analysis.

Domains	Principal codes	Subcodes
General nurse feedback	General experience	—^a^
	Expectations	Expectations preimplementation, and fulfillment of expectations
	Overall satisfaction	—
	Evaluation of study activities	Satisfaction with tasks
	Benefits	Perceived personal benefits
Evaluation of training received	Evaluation of training received	Level of preparedness posttraining, missing subjects, and suggestions for training
Evaluation of activities related to the study	Evaluation of study activities	Initial appointments, monitoring calls, nonadherence calls, help requests, revision of nurse dashboard, registering tasks in nurse dashboard, supervision meetings, and difficulties
Feasibility of incorporating CONEMO in primary care	Feasibility of scaling up CONEMO	—
	Incentives	Types of incentives desired
	Suggestions for the project	—

^a^For some principal codes, further subdivision did not seem to be beneficial, therefore no subcodes were created.

## Results

### Participants’ Characteristics

#### Patients

Among the 29 patients, who responded to the interviews, the mean age was 60 years (SD 9.6, range: 47-85 years) and 69% (N=29) were women. Almost half of the patients were diagnosed with both diabetes and hypertension (45%, N=29), whereas 31% were diagnosed with solely diabetes and 24% with solely hypertension. Less than half of the patients, 45% (N=29), reported having experience in using a smartphone. For an overview of the demographic variables of the patients, see Multimedia Appendix 6.

#### Nurses

All nurses who participated were women, the mean age was 38 years (SD 6.2, range: 31-46 years) and the mean time working in the health care center where they were currently employed was 6.6 years (SD 5.3, range: 3-16 years). All nurses had higher education and were licensed in nursing, 1 additionally had a PhD degree, and most had previous experience with using smartphones (83%, N=6) or tablets (67%, N=6). In 1 of the 2 health care centers, 1 nurse monitored 7 patients, whereas in the other center, 5 nurses monitored 10 patients, 2 patients each, who participated in the second pilot study.

### Patients’ Experience With CONEMO

#### Perceived Health Benefit of CONEMO

Almost all patients (93%; if not mentioned otherwise, the denominator for all frequencies in this paper is the total number of patients, N=29) said that CONEMO helped them emotionally. Most patients felt that after having used CONEMO, they were calmer, more cheerful, enthusiastic, motivated, and less worried and stressed out. Although more than half of the patients (59%) mentioned doing new activities or having picked up activities they have not been doing in a while, one-third (34%) attributed their improvement in emotional well-being to being more active. People did not only increase healthy activities, but also pleasant activities, such as reading a book, visiting friends and family, playing soccer with other people, or walking the dog. As 1 patient expressed it:

[CONEMO] has given me the opportunity to rediscover what we have in life.

Patient 32474, female, 65 years, quote #1; see Multimedia Appendix 7 for original quotes in Spanish and previous smartphone experience.

One patient explained that being more active distracts oneself from the negative thoughts and therefore improves psychological health.

Another important factor related to the perceived improvement was that CONEMO gave them the feeling of not being alone (34%). CONEMO was viewed as a *conscious friend*, as a *personal psychologist*, or just as *someone* that gave them advice, *someone* they did not have in their lives before, which made them feel not being *forgotten* about or *abandoned*, as they had previously assumed. Interestingly, 2 people (7%) also mentioned to have more self-esteem after having used CONEMO, as the following quote describes:

CONEMO, yes, it improved my emotional health because I felt that I mattered, that I have to allocate some time to myself and that no one matters more than me. I have been feeling better emotionally, freer. I felt that I did not have to depend on anyone and that I can do things for myself.

Patient 12406, female, 51 years, quote #2, Multimedia Appendix 7.

Some patients also valued positively the study safety procedures—which were not part of the intervention itself—to motivate patients to look for professional help, transfer them to mental health professionals within the health care system, and in cases of severe depressive symptoms, even helping them to receive treatment faster than usual (14%). Some patients felt that this also contributed positively to their psychological health. However, 2 people (7%) thought that the time of the intervention was not sufficient to accomplish groundbreaking improvements and other 2 did not feel that CONEMO improved their psychological health.

As many activities suggested by the intervention were healthy activities that people with NCDs could complete, some of the patients (34%) also felt that CONEMO helped them improve their physical health. For example, some mentioned having lost weight because of their healthier diet or others to have gained mobility because of increased exercising.

In addition, the majority of the patients mentioned changing their daily habits toward a healthier routine (62%). Most of them reported exercising more, eating healthier, taking the bike to buy groceries, walking or running more, and taking their medication more regularly. One person also described that CONEMO motivated her to go the doctor, whereas she had previously tried to avoid all doctor consultations if possible.

In terms of *other benefits perceived*, 6 patients (24% of 25) described CONEMO as helping them to organize their everyday routine better to do different activities. Some (20% of 25) also mentioned that through CONEMO they were more conscious about their chronic condition and paid more attention to their health and their body. CONEMO has also changed some of the patients’ relationships with their families and friends (16% of 25), for example, by implementing CONEMO’s suggestion of exercising in form of weekly family volleyball games.

#### Usability

Most patients encountered some kind of difficulty in using CONEMO or the smartphone (72%) and would have liked to receive more training. Some patients had problems remembering the explanations received from the nurse (14%). Although about half of the patients admitted that they did not know how to handle the technology in the beginning (48%), most patients with difficulties (71% of 21) explained that those generally occurred in the beginning and that, with time and practice, it got easier to use CONEMO and the smartphone. Furthermore, 14 patients (48%) mentioned having received help from others, such as family members, in using CONEMO. The great majority of the patients reported reviewing the guidebooks provided by the research team (90%) and found them helpful (74% of 23).

**Figure 1:**
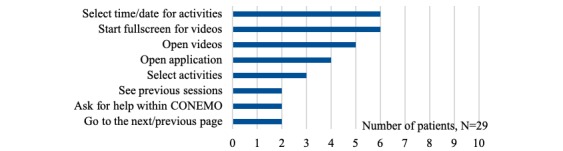
Difficulties using CONEMO.

**Figure 2:**
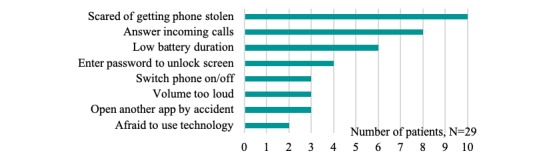
Difficulties with the smartphone.

The specific difficulties with CONEMO and the smartphone mentioned by the patients are described in Figures 1 and 2, respectively. Regarding CONEMO, most difficulties were experienced when selecting a time and date for scheduling an activity by changing the numbers using up and down arrows. Concerning difficulties using the smartphone itself, patients mostly mentioned the fear of getting it stolen.

Furthermore, many people experienced difficulties with the interactive tools used in this intervention, such as SMS, Android notifications, and dialogue pop-ups. The SMS served as a reminder for the appointments within this study, Android notifications informed patients that a new session had arrived, and dialogue pop-ups were designed to retrieve feedback about the completion of the activities through messages appearing on the screen soliciting a response from the patient. There were some technological and connectivity problems, for example, people did not receive the SMS in time. However, most people did not view or use the Android notifications (51% of 24) and opened CONEMO directly from the home screen (71% of 24) to see if new sessions had arrived, and some people did not know how to open the SMS or could only read them with the help of others (21% of 24). The idea behind the dialogue pop-ups—to give feedback about activity completion—was evaluated positively; however, some did not recall them (25% of 24) when asked about them.

#### Adherence to CONEMO

This section refers to the self-reported adherence to CONEMO in the interviews. Another publication by the LATIN-MH team addresses the quantitative adherence data of the pilot studies [57]. Adherence to CONEMO is defined here as (1) the amount of CONEMO sessions reviewed and (2) the amount of activities completed that were suggested by CONEMO. Regarding adherence to session review, around half of the patients who talked about it said they not only read all of the sessions of CONEMO (65% of 20) but also re-read numerous sessions (50% of 20). In addition, 2 people even re-read the whole intervention again before returning the smartphone to the research team. Some explained that they re-read sessions to better remember the content or because it served them as a reminder to do certain activities, especially in between sessions or at the end, when there were no new sessions available. On the contrary, some people also disclosed that they did not complete all the sessions (30% of 20). In terms of the adherence of activity performance, 9 people (39% of 23) indicated to have done all activities they selected during this intervention and other 9 (39% of 23) completed most of the activities. Thus, the great majority seemed to have been very adherent to the intervention. The activities with the highest completion rate were pleasant activities, such as going out meeting friends and family, and healthy activities, for example, going for a walk, whereas pending tasks, such as organizing the bills or paying them, seemed less frequent to be done. However, 5 (22% of 23) people did few or none of the activities.

The main barrier reported to performing the activities suggested by CONEMO was the users’ health status (36% of 14), such as limited mobility or memory problems. Time (29% of 14) and economic constraints (21% of 14) were also important barriers to doing activities. Other difficulties mentioned were the low motivation to do activities (9% of 23) as well as sudden changes in their plans because of external factors (14% of 14), for example, receiving a phone call or someone else needing assistance.

#### Satisfaction With CONEMO

Satisfaction refers to the statements that patients made about what they liked and disliked about the app and the sessions, comments that could infer satisfaction, as well as the patients’ perception about using technology for intervention delivery.

The majority of patients expressed great satisfaction with CONEMO (96% of 24), some did not make any statements regarding their satisfaction (17% of 29), and 1 was unsatisfied (4% of 24) because he felt that his health status was too compromised to follow all of the instructions. When the patients were asked what they liked most about the CONEMO app and its sessions, most appreciated the information and advice received (37% of 27), for example:

[…] its important guidelines [to do things one step at a time] instead of doing everything at once.

Patient 32433, female, 60 years, quote #3, Multimedia Appendix 7

Furthermore, 26% (of 27) of patients most liked the videos incorporated in CONEMO, 15% (of 27) most appreciated that they were reminded by CONEMO to follow their diet, to work out or follow their medication, and 11% (of 27) valued most the types of activities offered, as described by one of the patients:

In the [list of] activities there were things that caught my attention and that I would be able to do. This opened a whole new field for me of other activities I could do and that I had not done before.

Patient 32444, female, 64 years, quote #4, Multimedia Appendix 7

Furthermore, 2 patients even transcribed the whole intervention on their computer to be able to re-read it again after giving back the smartphone. Of note, one-third (33% of 27) of the patients especially valued being accompanied by CONEMO and the nurse and receiving monitoring phone calls from the research team, which were implemented as a safety procedure within the research rather than a treatment activity. These contacts transmitted a feeling of being cared for, of someone taking a special interest in them and their health, and of trusting them with a technological device:

I didn’t have anyone to talk to, so I watched the woman in the video, […].

Patient 12266, female, 76 years, quote #5, Multimedia Appendix 7

I was surprised and thought “what did I do to deserve something like this?”. And in addition, they gave me 10 Soles (Peruvian currency) to cover my transport. [...] This is the first time in my life that I have received this kind of attention. [...] No one has ever been concerned about me, but now there was someone there, who was worried about my health [...].

Patient 22131, male, 70 years, quote #6, Multimedia Appendix 7

**Figure 3:**
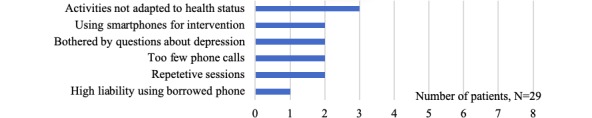
Aspects about CONEMO viewed negatively.

Many people (54% of 28) did not spontaneously express negative aspects about CONEMO*,* although it was specifically asked for. The statements that were made were very diverse, and there was not much consensus (see Figure 3).

Most of the patients (95% of 21) expressed a positive attitude toward using smartphones to deliver the intervention, and 48% (of 21) of people mentioned explicitly the advantage regarding the mobility, whereby the use of the smartphone replaced having to go to the health care center to make an appointment or to see a specialist. This potentially reduces the time spent on health consultation and could “*unclog the health care center’s environment* ” (Patient 22116, female, 52 years, Multimedia Appendix 7, quote #7), as 1 patient explained. In addition, 4 patients (19% of 21) also highlighted the practicality of being able to take the intervention with them, to re-do the sessions, and especially being able to choose the place and time to follow the intervention:

It is convenient, because we are not as close to the doctor or psychologist… Here, it does not matter, where you are, you can still use it.

Patient 32433, female, 60 years, quote #8, Multimedia Appendix 7

#### Suggestions for CONEMO

The suggestions that patients expressed to improve CONEMO were wide ranged and could be grouped into 2 categories: (1) suggestions for the content and design of the app and (2) communication with the research team.

**Figure 4:**
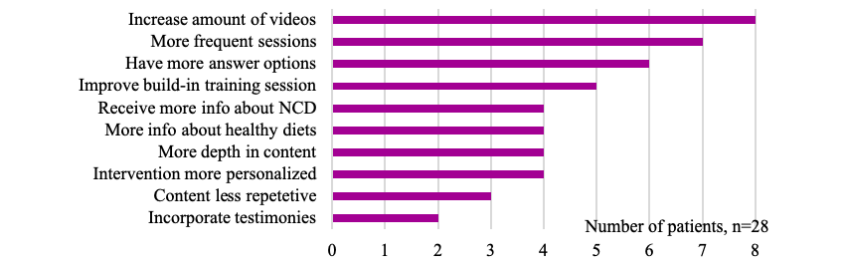
Suggestions for CONEMO content and design.

Regarding suggestions for the *content and design of the app*, most patients suggested increasing the duration of the intervention (68% of 28). The other suggestions are displayed in Figure 4 *.* In terms of preference of information delivery, if given the option, most people would prefer to be able to hear the content read to them via an audio (43% of 28) or to have both audio and text (36% of 28), whereas others prefer to only read the text (21% of 28).

In terms of the *communication with the research team*, patients also desired more. Some patients suggested adding group activities with all the patients and trainings or lectures of different topics (14% of 28). Others would have liked to receive personal face-to-face appointments with the research team for depression monitoring instead of phone calls or specialized psychological care via telephone or audio messages (11% of 28).

#### Evaluation of the Nurse Support

Most patients were satisfied with the nurses’ explanations of how to use CONEMO (59%); however, some patients mentioned that it was still difficult to understand (14%) or that they were not able to use CONEMO at first, directly after the training (17%). Various people said something similar to the following:

The nurse explained well, it is just that maybe I am stupid or something, because when I got home, I did not know how to use it.

Patient 12073, male, 66 years, quote #9, Multimedia Appendix 7

However, some of the patients who were accompanied by a nurse from the health system perceived the nurse as being in a hurry during the training (10%) or without interest in explaining the intervention (7%). Comparing both pilot studies, 80% (of 15) of the patients who were accompanied by the hired nurse viewed the training session as useful, with a positive knowledge transfer, whereas only 50% (of 14) of patients monitored by a staff nurse expressed either positive or neutral comments about the quality of the training.

**Figure 5:**
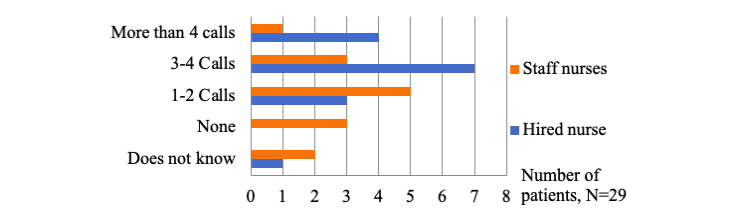
Quantity of contact with nurses.

On the basis of the study protocol, all patients should have received at least 2 phone calls by the nurse to see if there are any difficulties with CONEMO and additional calls depending on their adherence and difficulties. Evaluating the quantity of contacts, when working with a hired nurse, most of the patients reported having received between 3 and 4 phone calls from the nurse (53% of 15) during the intervention, whereas most patients accompanied by staff nurses reported to have received 1 to 2 calls (36% of 14; see Figure 5).

Most of the patients in both pilot studies viewed the phone calls as positive (81% of 26) because the nurses helped them to be adherent to the intervention (19% of 26), helped them with their difficulties using CONEMO (19% of 26), paid attention to their health (15% of 26), helped with other problems (8% of 26), and showed the patients that she cared (15% of 26).

Through *help requests* patients could solicit tech support from the nurse. Most of the patients stated they did not use the help request button within CONEMO (68% of 28), some because they claimed to not have needed it and others because they felt embarrassed, did not remember this function, or thought the nurse would be busy anyway. Some patients also asked for help by error (7% of 28). In addition, 4 people (14% of 28) expressed that they did ask for help and received it, whereas other 3 people stated that they had pressed the button but that no one had called them.

Regarding suggestions for improving the nurse-support, almost half of the patients would have liked to receive more phone calls (44% of 27), and ideally a space to talk about their emotional state, instead of only receiving technical support from the nurse. Others suggested amplifying or improving the quality or depth of the training to feel more confident in using CONEMO (11% of 27).

A summary of the main results retrieved from the patients’ interviews can be found in Table 3.

**Table 3:** Summary of main results—patients.

Domain	Main results
Perceived health benefit of CONEMO	Almost all patients perceived improvements in their mental health and some also in their physical health after using CONEMO.
	The majority felt more active after using CONEMO, having mostly increased pleasant and healthy activities.
	CONEMO was also viewed as a companion, which made them feel less alone.
Usability	Initially, most patients encountered difficulties in using CONEMO or the smartphone and with using SMS, Android notifications, and dialogue pop-ups; however, many technological issues also emerged around those elements, which is why the distinction of those two is not completely clear.
	Some patients received help from family members or reviewed the guidebooks, and most patients felt that these difficulties subsided with time and practice.
Adherence to CONEMO	Self-reported adherence was high, most patients completed all sessions and the majority completed most or all activities advised by CONEMO.
	The most important barriers to completing the outside-app activities were the patients’ health status, time, and economic constraints.
Satisfaction with CONEMO	Satisfaction with CONEMO was high, as it was with using smartphones for intervention delivery.
	Features most appreciated were the advice provided, the videos and types of activities suggested, as well as the monitoring calls received by the nurse.
	The few critiques were directed at the type of activities suggested by CONEMO and that the sessions were repetitive.
Suggestions for CONEMO	Patients suggested increasing the duration of the intervention, the amount of videos and frequency of sessions and improving the in-build training session.
	Many patients also desired a more frequent interaction with the nurses.
Evaluation of nurse component	Although most praised the explanation from the nurses, some still experienced difficulties afterward, and therefore, they suggested improving the training.
	The monitoring calls were viewed as positive and helpful to increase adherence and resolve difficulties.

### Nurses’ Experience Supporting CONEMO Users

#### General Nurse Feedback

Most nurses (5/6) felt that CONEMO was an innovative and useful intervention. They thought, it was a good idea, something different, an opportunity to talk more to the patients and something beneficial to them. Some nurses reported that participating in this project benefited themselves as well because they took part in improving the patients’ health (3/6) and gained experience participating in a research project (1/6). However, most nurses (5/6) mentioned having had difficulties in consolidating their CONEMO activities with their workflow and felt that this increased their workload. The difficulties mentioned were related to the fact that before implementation, the health care centers’ management agreed to provide the nurses with reserved hours to participate in the intervention; however, because of logistical and administrative constraints, once the study started, the nurses had to accommodate these additional activities in their daily routine without adjustments to their existing workload, as 1 nurse describes:

At the beginning, I found it tedious, because we did not have protected hours for this task. I took on more [tasks], because I had more free time. [...] When the [chief] doctor saw that we were having trouble, she said that when we were in the clinic, we should ask the auxiliary nurse to cover for us for a moment [to do our tasks related to CONEMO].

Nurse 306, female, 38 years, quote #10, Multimedia Appendix 7

All nurses felt strongly that the implementation of CONEMO including nurse-support must imply strictly reserved work hours to be executed as expected.

#### Evaluation of Training Received

Most nurses (5/6) stated that the training they had received was appropriate. However, 2 of them felt they were not able to remember all the procedures when the study started because of a lack of practice or a lack of concentration during the training sessions.

[I learned] about 80% [of the procedures] because it was difficult to stay focused during the training, due to the number of tasks that we had [to do] in the health care center.

Nurse 304, female, 33 years, quote #11, Multimedia Appendix 7

Regarding the study technology, 3 of the 6 nurses reported that it was more difficult to use the nurse dashboard in the tablet to monitor their patients than to learn and explain how to use CONEMO, and they recommended increasing the time to practice during the training. Of note, the training of the hired nurse was a lot less time intensive compared with the training of the nurses from the public health care system because attendance fluctuated and it was difficult to gather everyone at the same time, maintain their concentration and motivation, and have sufficient time per training session to conduct complete practice runs before the nurses had to leave again.

#### Evaluation of Activities Related to the Study

To complete their tasks, nurses were supposed to use the nurse dashboard to view and log their tasks as well as monitor their patients. Only 2 nurses revised the dashboard regularly, every other day. The other 4 only revised the dashboard 2 or 3 times during the 2 to 3 months they were monitoring patients because they did not have enough time (3/6), they forgot (1/4), or because the tablet was not always accessible to them (1/4) as it was stored in the director’s office.

One of the most important responsibilities of the nurses was the initial appointments in which they trained patients on how to use CONEMO. Most nurses (4/6) emphasized that their patients had some difficulties in understanding the technology, but that they were able to explain it again afterward or that the patients received help from their family members. Some nurses (2/6) had difficulties during the training because they themselves forgot how certain things worked, and another 1 reported time constraints that forced her to complete it as fast as possible.

Nurses were also supposed to complete monitoring calls; ask their patients if they had difficulties in using CONEMO; motivate them, if needed, to increase adherence; and answer help requests. Half of the nurses made the calls by their own initiative, whereas the other 3 did not complete the calls unless they were reminded to do so by the clinical supervisor. In addition, some of the nurses had great difficulties in reaching some of their patients (2/3).

Supervision meetings led by a psychologist were supposed to be conducted on a weekly basis. In 1 health care center, the nurse was very comfortable with the supervision because she felt the research team addressed her doubts. However, at the other health care center, nurses felt pressured to have those supervision meetings (4/5) mainly because they felt overwhelmed because of their workload:

You, [the research team], were not the problem, but the work burden we have here. We just could not do it, it was pretty uncomfortable because we did not have the time to receive you as we should have. [...] It felt like “another thing” that I have to do.

Nurse 304, female, 33 years, quote #12, Multimedia Appendix 7

#### Feasibility of Incorporating CONEMO in Primary Care

Considering the possibility of scaling up CONEMO plus nurse-support, all nurses identified the lack of time to perform the related activities as the main problem for its implementation within the health care system. All nurses considered it necessary that the activities of the nurse-support would be included as part of the monthly schedule and paid work hours, a decision that depends on the administration of the Social Security System:

You would have to separate [CONEMO] as an independent program because here each nurse is responsible for one program. It cannot interfere with other activities.

Nurse 301, female, 38 years, quote #13, Multimedia Appendix 7

**Table 4:** Summary of main results—nurses.

Domain	Main results
General nurse feedback	The intervention was viewed as useful, beneficial, and an opportunity to talk more to their patients. However, most nurses had difficulties to consolidate the study activities with their daily work routine.
Evaluation of training received	The training was viewed as appropriate, although most nurses had some difficulties because of lack of concentration and fluctuating attendance.
Evaluation of activities related to the study	Most nurses experienced some difficulties conducting their tasks reliably, especially concerning the review and registry of activities in the dashboard, conducting monitoring calls, and participating in supervision meetings.
Feasibility of incorporating CONEMO in primary care	The activities related to CONEMO would have to be part of their paid work hours instead of being additional tasks. Some nurses also considered incentives as a possibility to increase the feasibility to scale up.

One factor related to this issue, which inhibited the nurses from being adherent to the intervention, was their job insecurity (5/6): Each nurse is required to meet a productivity goal and if it is not achieved, their employment contract may not be renewed again.

When nurses were asked if incentives could improve their performance regarding to CONEMO activities, 3 of 6 nurses stated that they would consider incentives, such as a course or regular meals; however, one of them explained that her main interest in participating was based on the perception that CONEMO benefits her patients. Other 2 nurses said that an incentive might motivate their colleagues but they themselves are uncertain if they would be part of the CONEMO nurse-support again because of their personal time constraints, and 1 stated she would not participate in the intervention again, even if she would get paid.

The results for the nurses are summarized in Table 4.

## Discussion

### Principal Findings

In this study, we aimed to explore the experience of primary care patients and nurses with CONEMO, a technology-driven, nurse-supported intervention to reduce depressive symptoms in people with diabetes, hypertension or both, to derive lessons informing future adaptations, and to assess the feasibility of using this technology in LMIC settings. Almost all patients felt that CONEMO helped them to improve their mental health by being more active, increasing their self-esteem, and making them feel valuable. Many patients also experienced improvements in their physical health related to healthier diets and more physical activity. The self-reported adherence to this intervention was relatively high. Although most people reported some difficulties with the technology in the beginning and suggested amplifying the training, the majority felt that it got easier with time. Using smartphones for intervention delivery was generally seen as practical and time saving. The main recommendations were to include more sessions and videos, to increase personal contact, and to make it more personalized. Most patients viewed the nurse-support as positive, although the evaluation of the support of the hired nurse was more frequently positive than of the staff nurses. The majority of staff nurses participating in the pilot study felt that the intervention was valuable for patients but that the activities of CONEMO conflicted with their daily work routine.

### Reflections and Comparison With Previous Work

A growing body of research is aimed at capitalizing on the expansion of technology and its potential to introduce newer vehicles to target mental health, suggesting potential effectiveness of Web-based, text-messaging, and telephone support interventions [77]. However, few studies are being conducted in LMIC settings [78], which makes our studies highly relevant.

Multimorbidities, defined as having 2 or more diseases, including NCDs and mental conditions, constitute a growing health threat, especially in LMICs. However, most available treatments usually only focus on 1 health condition instead of approaching multimorbidities in a more integrative way [79,80]. Therefore, cost-effective treatments that address multiple conditions are highly needed, and future research should be aimed at testing the effectiveness of mHealth interventions targeting multiple conditions. Nevertheless, in technological interventions designed for people with multiple health conditions, such as CONEMO, special consideration needs to be put on the specific target population. For example, one challenge, especially in behavioral activation interventions, could be balancing the activity suggestions, considering the demand of physical mobility to complete them, so that people with more compromised health statuses can still participate, without feeling frustrated. In general, but especially in LMIC settings, economic constraints also have to be considered when creating activity selections. Other studies have also recommended that researchers be aware of additional challenges of people with chronic conditions, such as limited mobile literacy [81], which is in line with our findings. Although our study has shown satisfactory results, usability could be optimized by improving and amplifying training, considering the large proportion of people in this population, who are unfamiliar with the devices.

Although most people found the guidebooks useful and argued that the nurses generally explained well how to handle the technology, the majority reported some kind of difficulty in the beginning and suggested extending or improving the training. Although many difficulties will most likely subside as smartphones become ubiquitous, some changes to the design of CONEMO could also improve usability. Although most patients did not provide more information about how the training or design could be improved, one hypothesis was that a more realistic demonstration (using an in-build training session close to the actual CONEMO sessions) as well as a training video could be helpful to both nurses and patients. This way, the training would be more structured and more standardized over the nurses and, furthermore, would give the patient the opportunity to practice twice, while still being with the nurse: first, when conducting the training session and second, when completing the first real session. Nevertheless, in this study, as well as in others [82-86], there seems to be a natural learning curve, which for most patients resulted in the ability to use CONEMO. For some people, this learnability curve was intensified by receiving help from other family members. This suggests, on one hand, that reading about how to use the smartphone app seems to be less effective in this population than hearing the explanations from other people and receiving demonstrations of how to do it and, on the other hand, that friends or family members frequently become involved to some extent in the intervention delivery.

Although this intervention was created specifically for people with diabetes, hypertension or both, some of the results could also be relevant for other mental health interventions with different populations. Other studies suggest that key features of acceptable smartphone health apps seem to be self-help suggestions and feedback to the patients’ responses, they should be easy to use (*foolproof*), and not take too much time (maximum 10 min per single usage) [39,49]. A study by Baker et al [46] established 16 recommendations for developing mental health smartphone apps, of which 12 seem to apply to CONEMO, for example, encouraging non-technology-based activities, recording the patients’ behaviors, having a log of patients’ app use, applying reminders to engage them, and using a simple and intuitive interface. Other studies from high-income countries also indicate that the effectiveness, motivation, or adherence is higher when the health app is easy to use [39,48,49], is individually tailored [46], and includes human interaction [48,50-52]. Although most studies regarding depression apps where completed in high-income countries [39], our results appear to be in line with such previous findings.

Nevertheless, some of our interactive features showed low utility, and some technological problems were encountered. For example, most people did not check the Android notifications to see if a new session was available. It is plausible that those notification symbols were too small for this population, considering their average age. Another hypothesis could be that considering most people were novice users, who had not yet integrated the smartphone into their everyday lives and, hence, only used the smartphone for one app—CONEMO—they would not be interested in other Android notifications of the default apps already installed on the smartphone. Therefore, it might have appeared easier to open the one app of interest directly from the home screen. The utility of the other interactive elements, such as SMS and dialogue pop-ups, was difficult to evaluate given the technological problems experienced. Considering that those difficulties were not encountered during the testing phase and some could be related to signal changes in the field, formative research for large studies including technological platforms appears to be of high importance.

Another important consideration about health apps is related to the setting with special emphasis on crime rate. Given the perception of insecurity and fearing a possible robbery, many patients were reluctant to borrow a smartphone from the research team. This fear could be reduced by explaining that in case of a robbery patients would not be charged for the phone. However, as a consequence, many people did not use it as a *mobile* device, outside of their homes. This suggests that the socioeconomic conditions related to security can have an effect on the user’s experience with mHealth interventions. Nevertheless, after concluding the pilot studies, only 1 smartphone was stolen and 1 was not returned, constituting a loss of 6%, which was a lot lower than expected. Considering scalability, installing CONEMO on the patients’ own phones if available and, hence, reducing the perceived responsibility for a borrowed phone could be one way to reduce this reluctance besides not holding patients accountable for a possible robbery or loss of the smartphone and covering those costs.

Evidence shows that in technology-only interventions, where people are left alone with mobile self-help interventions, participants are less adherent [37,50-52] and less motivated to engage in the program [87] than people who are accompanied by professionals or coaches or have some other kind of face-to-face interaction as part of the intervention. In this formative research, the nurse embodied this role. The majority of patients reported being highly satisfied with CONEMO, they valued the advice and information provided, but some patients especially appreciated the attention received within the study. This was not anticipated, considering that the nurses’ role was based on giving technological support, but apparently, their accompaniment was perceived as beyond that role. In line with this finding, other studies emphasized that supportive accountability increases the adherence to any intervention and the extent of influence is characterized by “the bond between the user and supporting individual, the sense of accountability, and the user’s perception of the supporting individual as possessing legitimacy and expertise” [50]. It is plausible to assume that the combination of technology with an in-person monitoring in these studies also influenced the slightly higher self-reported adherence rates of 65% to 78% and the low dropout rate of 12%, when compared with other similar studies, which found average adherence rates of 50% to 70% and dropout rates of 1% to 57% [88].

In addition to the nurse-support, CONEMO contained an *intermediate* feature between technology and a personal component, which is relevant to consider: the videos in the CONEMO sessions were narrated by a *real* person, who was maintained throughout the intervention. Although patients never met this person, some people felt a connection to her and imagined she was the *face behind the technology*, who talked directly to them, who monitored them, and who had a leading role steering this intervention. Therefore, some people felt a personal relationship to the woman in the videos.

The importance of the videos was also highlighted by the patients’ suggestion to incorporate more of them. However, when designing this type of intervention and considering the number of videos, developers need to prioritize and contemplate the size of the videos (especially in a fully functioning offline app), which influences the speed of installation versus, for example, relying on a stable internet connection to watch videos on the Web. Those technological considerations need to be in line with the research priorities.

Feasibility of working with nurses within the health system to monitor the patients was low because of the conflicting use of time with their daily activities. Especially given their job uncertainty, many nurses felt pressured. Other studies in a very similar context also identified workload and time constraints as the most important barriers to implementation, especially taking into account that the studies’ activities were additional to their existing tasks, without considering more paid time [89]. Therefore, when scaling up these types of task shifting interventions within the public health sector, specific resource allocation, such as workflow adaptations or incentives, have to be considered to overcome those barriers. It is also crucial to conduct cost-effectiveness studies and evaluate potential cost-savings to address organizational-level interests in implementing technology-based interventions and make correlated resource allocation more appealing to health care systems.

Adherence to the study activities can also be influenced by other factors specific to the health care center settings. For example, in our study, adherence seemed to be to some extent related to the attitudes of the nurses toward the study (positive attitude–higher adherence, and vice versa). This, in turn, could have been influenced by the management style of the health care center; the 5 nurses from 1 health care center who monitored 2 patients each were all assigned by their superiors to this study, although most were initially reluctant to participate. The one nurse with the highest workload—monitoring 7 patients—who had experience participating in different health programs, was asked by her superior beforehand, and she had the most positive attitude toward the intervention, was highly motivated, and ultimately was less affected by the workload of CONEMO. Therefore, although some health care center managers were initially enthusiastic about the study, important factors, distinct for each health care center, such as staff management, work climate, and conditions, could also influence implementation success. Furthermore, although it is ideal to find motivated nurses enthusiastic with this intervention, this may not be feasible across all of Lima or Peru, presenting a challenge to scalability; thus, structured resources should be put in place.

LATIN-MH benefited from this detailed formative phase conducted across 2 distinct health care provider systems in Lima, a large Latin American capital. Conducting qualitative formative research stages to shape mental health interventions has also been beneficial for other studies [90]. In our case, this approach set the foundation for the modifications and adaptations of the intervention to build an app tailored to the patients’ needs before deploying a complex intervention such as CONEMO in a large randomized controlled trial, as recommended by other studies [45,46,50].

### Limitations

Although this study has provided important insights in the evaluation of an mHealth intervention for people with NCDs and depressive symptoms, this study also had some limitations. For example, although all patients were provided with the same intervention and number of sessions, the intervention uptake might not have been the same for all of the patients, depending on their adherence to review the sessions. However, the number of patients included in this study was sufficient to signal positive and negative experiences with the CONEMO intervention [58]. Therefore, we feel confident that we have sufficient information to improve the future design of CONEMO for the clinical trial.

Another limitation of this study is related to the fact that for the first pilot study in the MINSA hospital, a nurse was hired, whereas in the second pilot study in the Social Security System, nurses from the health system accompanied the patients. Therefore, although the perspectives of patients from both public health systems were obtained, only the perspectives of the nurses within the Social Security System were analyzed. Furthermore, it is plausible that the patients’ experience differed depending on what type of nurse accompanied them throughout the intervention beyond what has been identified and described here. However, conducting this second pilot study with staff nurses was extremely beneficial for the study purposes to explore feasibility of working with staff nurses and understanding the challenges they encounter, which might be similar in other public health systems. Therefore, after the first experience, it was crucial to conduct a second pilot study with this aim.

Furthermore, the interviews were not recorded and, therefore, there is a possibility that some information from the participants was lost. However, the 2 people who conducted the interviews aimed at writing down the verbal statements literally and took notes simultaneously during some of the interviews, which were contrasted afterward and which showed high literal consistency. This makes us confident that the notes represent the participants’ statements reliably.

### Next Steps

Although generalizability is limited, all of these findings are relevant and crucial to inform the development of future complex interventions for mental health conditions, multimorbidity, and specifically for further designing and testing the efficacy of CONEMO under more controlled designs; indeed, CONEMO will be tested in 2 parallel randomized controlled trials. Other studies have demonstrated that mobile apps can effectively change behavior; however, considering the limited evidence, uncertainty of long-term effects and the predominance of those studies in high-income countries so far [39], the randomized controlled trial will give important insights regarding this type of interventions in LMIC settings.

Furthermore, this study is especially relevant considering the current mental health reform in Peru, where for the last 12 years, several efforts have been made aimed at improving the availability of free and universal access to mental health care for Peruvian citizens through a community-based approach, including primary health care centers [19,91]. In this effort, tasks are already being shifted toward personnel not specialized in the same field, integrating mental health care in a variety of different medical settings. This development creates an opportunity for scaling up cost-effective mHealth interventions with task shifting components in the public health care system.

### Conclusions

Smartphone apps constitute a potentially cost-effective opportunity in LMIC settings to overcome health system barriers and extend mental health care to large populations. On the basis of the experiences and opinions of the patients, it seems feasible to use this nurse-supported mHealth intervention for people with chronic diseases and comorbid depressive symptoms in Peru. CONEMO is perceived as helpful in improving mental health, but it requires context-specific adaptations, especially regarding the implementation of a task shifting approach within the public health care system; working with staff nurses using a task shifting approach within the public health system in Lima will only be feasible if the nurses’ time is protected for the program. Findings from this study will provide important information to develop a larger study focused on testing the effectiveness of this program on patients’ health and mental health outcomes.

## Appendix

Multimedia Appendix 1 Screenshot of CONEMO.

Multimedia Appendix 2 Interview guide—patients.

Multimedia Appendix 3 Interview guide—nurses.

Multimedia Appendix 4 Codebook—patients.

Multimedia Appendix 5 Codebook—nurses.

Multimedia Appendix 6 Demographic variables—patients.

Multimedia Appendix 7 Original quotes in Spanish with demographic information.

## Abbreviations

DALY: disability-adjusted life year
LATIN-MH: Latin America Treatment and Innovation Network in Mental Health
LMIC: low- and middle-income country
mHealth: mobile health
MINSA: Ministry of Health
NCD: noncommunicable disease
SMS: short message service
