# Supplementary figures and images for: Addressing Depression Comorbid With Diabetes or Hypertension in Resource-Poor Settings: A Qualitative Study About User Perception of a Nurse-Supported Smartphone App in Peru
Source: JMIR Ment Health. 2019 Jun 18;6(6):e11701. doi: 10.2196/11701 (PMC6604501; doi:10.2196/11701)

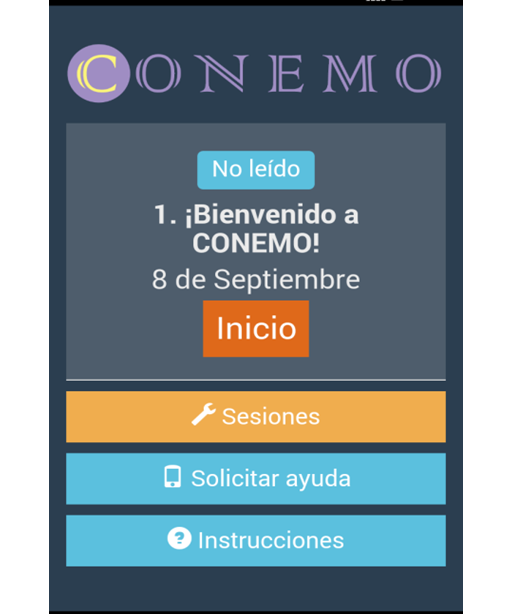

Supplement: Multimedia Appendix 1 [file mental_v6i6e11701_app1.png]
