# Supplementary material for: Addressing Depression Comorbid With Diabetes or Hypertension in Resource-Poor Settings: A Qualitative Study About User Perception of a Nurse-Supported Smartphone App in Peru
Source: JMIR Ment Health. 2019 Jun 18;6(6):e11701. doi: 10.2196/11701 (PMC6604501; doi:10.2196/11701)
Supplement: Multimedia Appendix 2 [file mental_v6i6e11701_app2.docx]

## **Multimedia Appendix 2: Interview Guide – Patients**

**FUP 2A: Guía de Entrevista CONEMO**

Fecha de la entrevista:
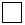

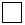

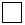

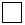

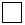

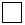

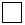

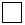


Código del participante:
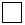

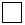

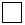

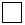

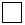

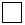


Código del entrevistador:
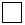

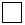


**1. Evaluación de la intervención CONEMO**

1. ¿Qué le parece la idea de usar celulares para ayudar a la gente a mejorar su estado de ánimo? (¿Por qué?)
2. ¿Cómo podría ayudar CONEMO a personas con enfermedades crónicas cómo diabetes mellitus o hipertensión?
3. ¿Cómo sintió que participar en este estudio afectó a usted?
4. ¿Cómo cree que CONEMO ayudó a su salud emocional?
5. ¿Cómo cree que CONEMO ayudó a su salud física?

**2. Evaluación de CONEMO**

1. ¿Qué es lo que más le gustó de CONEMO?
2. ¿Qué es lo que menos le gustó de CONEMO?
3. ¿Qué es lo que le gustó de las sesiones?
4. ¿Qué es lo que no le gustó de las sesiones?
5. ¿Qué dificultades tuvo usando CONEMO?
6. ¿Sintió que en algún momento CONEMO no funcionó correctamente?
7. ¿Qué le parece si además de leer el texto, usted lo podría escuchar en una grabación? (¿Por qué?)
8. ¿Cómo se podría mejorado CONEMO?

**3. Evaluación del celular**

1. ¿Qué es lo que le gustó del celular?
2. ¿Qué es lo que no le gustó del celular?
3. ¿Qué dificultades tuvo con el celular? *(Batería, notificaciones, contestar el teléfono, abrir mensajes, etc.)*
4. ¿Aparte de usted, hubo otra persona que usó el celular? *(Si sí, específica quién y para qué se usó)*

**4. Evaluación de las entrevistas**

*Me gustaría que recuerde cuando le invitaron a participar en el estudio.*

1. ¿Qué le pareció esta conversación? *(¿Le gustó? ¿No le gustó? ¿Cómo se sintió?)*
2. ¿Qué otra cosa le gustó de la entrevista?
3. ¿Qué otra cosa no le gustó de la entrevista?

**5. Evaluación de la intervención de la enfermera**

1. ¿Cómo le fue en la cita de entrenamiento con la enfermera?
2. ¿Hay algo que le hubiera ayudado más en esta cita de entrenamiento?
3. Luego de la cita de inicio, ¿cuántas veces ha tenido contacto con la enfermera?
   - Cantidad: ______________
4. ¿Cómo le fue en los momentos en que se contactaron?
5. ¿Usted alguna vez pidió ayuda a la enfermera a través de CONEMO? (¿Por qué?)
   - (Dashboard: ____________________)
6. ¿Recibió la ayuda de la enfermera?
7. ¿Cree que hay algo que se podría mejorar en relación al contacto con la enfermera?

**6. Evaluación de las guías para el participante**

1. ¿Usted leyó la guía para el participante?
2. ¿Usted leyó la guía sobre el uso del celular?
3. Si 9a y/o 9b = sí: ¿Siente usted que las guías le ayudaron a utilizar CONEMO? *(¿Cómo? ¿Por qué?)*

**8. ¿Le gustaría decir algo más?**
