# Supplementary material for: Addressing Depression Comorbid With Diabetes or Hypertension in Resource-Poor Settings: A Qualitative Study About User Perception of a Nurse-Supported Smartphone App in Peru
Source: JMIR Ment Health. 2019 Jun 18;6(6):e11701. doi: 10.2196/11701 (PMC6604501; doi:10.2196/11701)
Supplement: Multimedia Appendix 3 [file mental_v6i6e11701_app3.docx]

## **Multimedia Appendix 3: Interview Guide – Nurses**

Fecha de la entrevista:
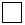

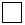

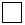

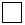

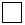

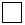

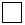

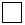


Código de la enfermera:
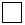

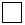

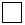

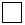

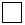

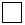


Código del entrevistador:
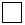

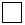


**Percepción y expectativas**

1. ¿Qué te pareció la experiencia de participar en este estudio? ¿Por qué?
2. ¿Al inicio, cuando les contamos de este proyecto, cuáles fueron tus expectativas acerca de tu rol en este estudio?
3. ¿Sentiste que estas expectativas se cumplieron? ¿Por qué?

**Entrenamiento**

1. Durante la primera etapa del proyecto, se le ofreció un entrenamiento para aprender las tareas que debían realizar y enseñarles cómo usar los teléfonos celulares y la página web en la Tablet. ¿Después de este entrenamiento, te sentiste preparada para hacer adecuadamente las tareas asignadas? (Ej. Entrenar al paciente, etc.)
2. Si no fue suficiente, preguntar: ¿por qué? ¿en qué tareas sentiste que no fue suficiente?
3. ¿Qué aspectos del entrenamiento que recibiste podrían o deberían mejorarse? Piensa, por ejemplo, en el método de enseñanza, el tiempo de entrenamiento, los materiales de apoyo, etcétera.

**Rol de la enfermera**

1. Nosotros te propusimos hacer distintas tareas. Ahora quisiera saber cómo te fue en cada una (*explorar siempre qué dificultades tuvo en cada una*):
   1. ¿Cómo te fue en las citas de entrenamiento con los pacientes?
   2. ¿Cómo te fue en las llamadas de monitoreo con los pacientes?
   3. ¿Cómo te fue en las llamadas de no-adherencia?
   4. ¿Cómo te sentiste al realizar llamadas por solicitudes de ayuda del paciente?
   5. ¿Cómo te fue con la tarea de revisar la Tablet con tus pacientes todos los días?
   6. ¿Cómo te fue al registrar en la tablet las actividades que hiciste?
   7. ¿Cómo te sentiste con las llamadas y reuniones con el equipo de investigación para ver cómo andaba todo?
2. ¿Qué dificultades tuviste participando en este estudio?
3. En general, ¿cómo te sentiste con la cantidad de tareas asignadas?

**Motivación**

1. ¿Qué cosas positivas has obtenido al participar de esta experiencia? (Por ejemplo, a nivel personal, laboral, etcétera).
2. ¿Sientes que los pacientes que participaron del estudio se beneficiaron de alguna manera? ¿Por qué?
3. ¿Es posible incorporar en su rutina las tareas que le pedimos hacer como parte de CONEMO? ¿Por qué? ¿Cómo podría hacerse para lograrlo? (*Explorar si son cambios en el sistema, etc.*).
4. Si diéramos incentivos a las enfermeras, ¿qué tipo de incentivos te podrían animar para hacer esta tarea a largo plazo?
5. ¿Si diéramos incentivos económicos, existe alguna figura en la cual son permitidos?
6. ¿Qué sugerencias tienes para mejorar el proyecto?
7. Si pudieras volver a elegir, ¿volvería a participar?
