# Supplementary material for: Addressing Depression Comorbid With Diabetes or Hypertension in Resource-Poor Settings: A Qualitative Study About User Perception of a Nurse-Supported Smartphone App in Peru
Source: JMIR Ment Health. 2019 Jun 18;6(6):e11701. doi: 10.2196/11701 (PMC6604501; doi:10.2196/11701)
Supplement: Multimedia Appendix 4 [file mental_v6i6e11701_app4.docx]

## **Multimedia Appendix 4: Codebook Patients**

| **1.** | **Satisfaction with CONEMO** |
| --- | --- |
| 1.1 | Using smartphones for intervention delivery |
| 1.2 | General feedback |
| 1.3 | Things most liked about CONEMO |
| 1.4 | Things least liked about CONEMO |
| 1.5 | Things most liked about the sessions |
| 1.6 | Things least liked about the sessions |
|  |  |
| **2.** | **Perceived health benefit of CONEMO** |
| 2.1 | Perceived benefit on psychological health |
| 2.2 | Perceived benefit on physical health |
| 2.3 | Other benefits perceived |
|  |  |
| **3.** | **Evaluation of the CONEMO content** |
| 3.1 | Written content |
| 3.2 | Videos |
| 3.3 | Within app instructions |
|  |  |
| **4.** | **Usability of CONEMO** |
| 4.1 | General comments about usability |
| 4.2 | Difficulties with CONEMO usage |
| 4.3 | Functioning of CONEMO |
| 4.4 | Help from others |
|  |  |
| **5.** | **Adherence to CONEMO** |
| 5.1 | Revision of sessions |
| 5.2 | Performing activities |
| 5.3 | Difficulties (to perform activities) |
|  |  |
| **6.** | **Design** |
| 6.1 | Design of CONEMO |
| 6.2 | Procedures of the intervention |
|  |  |
| **7.** | **Interactive tools (notifications, dialogue pop-ups and SMS)** |
|  |  |
| **8.** | **Suggestions for CONEMO** |
| 8.1 | Suggestions to improve CONEMO |
| 8.2 | Preference of how to receive information |
|  |  |
| **9.** | **Duration and frequency of the intervention** |
| 9.1 | Duration of intervention |
| 9.2 | Frequency of sessions |
|  |  |
| **10.** | **Evaluation of smartphone** |
| 10.1 | Things most liked about the smartphone |
| 10.2 | Thinks least liked about the smartphone |
| 10.3 | Difficulties with smartphone usage |
|  |  |
| **11.** | **Other usage of CONEMO** |
| 11.1 | Other people using the smartphone |
| 11.2 | Other effects perceived |
| 11.3 | Internet speed |
|  |  |
| **12.** | **Guidebooks** |
| 12.1 | Revision of guidebooks |
| 12.2 | Utility of guidebooks |
|  |  |
| **13.** | **Evaluation of nurse component** |
| 13.1 | General perception |
| 13.2 | Evaluation of training |
| 13.3 | Quantity of contacts |
| 13.4 | Evaluation of contacts |
| 13.5 | Help requests |
| 13.6 | Suggestions for the nurse component |
|  |  |
| **14.** | **Interviews with the research team** |
