# Supplementary material for: Addressing Depression Comorbid With Diabetes or Hypertension in Resource-Poor Settings: A Qualitative Study About User Perception of a Nurse-Supported Smartphone App in Peru
Source: JMIR Ment Health. 2019 Jun 18;6(6):e11701. doi: 10.2196/11701 (PMC6604501; doi:10.2196/11701)
Supplement: Multimedia Appendix 5 [file mental_v6i6e11701_app5.docx]

## **Multimedia Appendix 5: Codebook Nurses**

| **1** | **General experience** |
| --- | --- |
|  |  |
| **2** | **Expectations** |
| 2.1 | Expectations preimplementation |
| 2.2 | Fulfillment of expectations |
|  |  |
| **3** | **Evaluation of training received** |
| 3.1 | Level of preparedness post-training |
| 3.2 | Missing subjects |
| 3.3 | Suggestions for training |
|  |  |
| **4** | **Evaluation of study activities** |
| 4.1 | Initial appointments |
| 4.2 | Monitoring calls |
| 4.3 | Nonadherence calls |
| 4.4 | Help requests |
| 4.5 | Revision of Nurse Dashboard |
| 4.6 | Registering tasks in Nurse Dashboard |
| 4.7 | Supervision meetings |
| 4.8 | Difficulties |
| 4.9 | Satisfaction with tasks |
|  |  |
| **5** | **Benefits** |
| 5.1 | Perceived personal benefits |
| 5.2 | Perceived benefits for patients |
|  |  |
| **6** | **Feasibility of scaling-up CONEMO** |
|  |  |
| **7** | **Incentives** |
| 7.1 | Types of incentives desired |
| 7.2 | Regulation of economic incentives |
|  |  |
| **8** | **Suggestions for the project** |
|  |  |
| **9** | **Overall satisfaction** |
