# Supplementary material for: Addressing Depression Comorbid With Diabetes or Hypertension in Resource-Poor Settings: A Qualitative Study About User Perception of a Nurse-Supported Smartphone App in Peru
Source: JMIR Ment Health. 2019 Jun 18;6(6):e11701. doi: 10.2196/11701 (PMC6604501; doi:10.2196/11701)
Supplement: Multimedia Appendix 6 [file mental_v6i6e11701_app6.docx]

## **Multimedia Appendix 6: Demographic Variables for Patients**

| **Demographic Variables** | | **n (N=29)** | **%** |
| --- | --- | --- | --- |
|  |  |  |  |
| **Age** |  |  |  |
|  | 41-60 | 15 | 52% |
|  | >=61 | 14 | 48% |
| **Sex** |  |  |  |
|  | Male | 9 | 31% |
|  | Female | 20 | 69% |
| **Diagnosis** |  |  |  |
|  | Diabetes | 9 | 31% |
|  | Hypertension | 7 | 24% |
|  | Diabetes + Hypertension | 13 | 45% |
| **Education level** |  |  |  |
|  | Incomplete primary school | 2 | 7% |
|  | Complete primary school | 3 | 10% |
|  | Incomplete high school | 8 | 28% |
|  | Complete high school | 5 | 17% |
|  | Complete technical education | 3 | 10% |
|  | Incomplete technical education | 6 | 21% |
|  | Incomplete university education | 2 | 7% |
| **Family Income (per month)** |  |  |  |
|  | < S/. 750 (< ≈ 226 US$) | 3 | 10% |
|  | S/.750 – S/.1500 (≈ 226 – 452 US$) | 9 | 31% |
|  | S/.1501 – S/.2250 (≈ 453 – 679 US$) | 5 | 17% |
|  | S/.2251 – S/.3000 (≈ 679 – 906 US$) | 5 | 17% |
|  | More than S/.3000 (≈ 906 US$) | 4 | 14% |
|  | Do not know | 3 | 10% |
| **Have a cellphone** |  |  |  |
|  | No | 5 | 17% |
|  | Yes | 24 | 83% |
| **Have a smartphone (of those who have cellphone)** |  |  |  |
|  | No | 7 | 29% |
|  | Yes | 17 | 71% |
| **Know how to use a smartphone** |  |  |  |
|  | No | 16 | 55% |
|  | Yes | 13 | 45% |
