# Supplementary material for: Addressing Depression Comorbid With Diabetes or Hypertension in Resource-Poor Settings: A Qualitative Study About User Perception of a Nurse-Supported Smartphone App in Peru
Source: JMIR Ment Health. 2019 Jun 18;6(6):e11701. doi: 10.2196/11701 (PMC6604501; doi:10.2196/11701)
Supplement: Multimedia Appendix 7 [file mental_v6i6e11701_app7.docx]

## **Multimedia Appendix 7: Original quotes in Spanish with demographic information**

| **No. of quote** | **ID of patient / nurse** | **Sex** | **Age** | **Knew how to use smart-phone before** | **English translation** | **Original quote in Spanish** |
| --- | --- | --- | --- | --- | --- | --- |
|  |  |  |  |  |  |  |
| 1 | Patient 32474 | F | 65 | Yes | “[CONEMO] has given me the opportunity to rediscover what we have in life” | “Me ha dado la oportunidad de redescubrir lo que tenemos en la vida.” |
| 2 | Patient 12406 | F | 51 | Yes | “CONEMO, yes, it improved my emotional health because I felt that I mattered, that I have to allocate some time to myself and that no one matters more than me. I have been feeling better emotionally, freer. I felt that I did not have to depend on anyone and that I can do things for myself.” | “CONEMO sí, me ayudó en mi salud emocional porque sintió que YO valgo, que tengo que darme un tiempo y que nadie vale más que yo. Me he sentido mejor emocionalmente, más libre. Me sentí que no tenía que estar sujeta a nadie y que me puedo valer por mí mismo.” |
| 3 | Patient 32433 | F | 60 | Yes | “its’ important guidelines […to do things one step at a time] instead of doing everything at once” | “Una gran ayuda, porque te da pautas importantes para seguir y no hacerlo de golpe.” |
| 4 | Patient 32444 | F | 64 | Yes | “In the [list of] activities there were things that caught my attention and that I would be able to do. This opened a whole new field for me of other activities I could do and that I had not done before.” | “En las actividades hay cosas que me llamaron la atención que podía hacer. Esto me abrió un campo de otras actividades que podía realizar y que no hacía.” |
| 5 | Patient 12266 | F | 76 | No | “I didn’t have anyone to talk to, so I watched the woman in the video, […]. | “No tenía con quien hablar, entonces miraba la Srta. del video […].” |
| 6 | Patient 22131 | M | 70 | No | “I was surprised and thought ‘what did I do to deserve something like this?’. And in addition, they gave me 10 Soles (Peruvian currency) to cover my transport. [...] This is the first time in my life that I have received this kind of attention. [...] No one has ever been concerned about me, but now there was someone there, who was worried about my health [...].” | “Me sorprendí, pensé ‘qué hice yo para merecerme algo así’. Además me dieron 10 Soles para mi pasaje. [...] Es la primera vez en mi vida que he recibido este tipo de atención. [...] Nadie se ha preocupado por mi, pero ahora había alguien que se preocupó por mi salud [...].” |
| 7 | Patient 22116 | F | 52 | Yes | [This potentially reduces the time spend on health consultation and could] “unclog the healthcare center’s environment.” | “Es bueno para desatorar el ambiente de los centros de salud.” |
| 8 | Patient 32433 | F | 60 | Yes | “It is convenient, because we are not as close to the doctor or psychologist… Here, it does not matter, where you are, you can still use it.” | “Es conveniente porque a veces no estamos tan cerca al médico o psicólogo... Aquí no importa, igual lo puedes usar.” |
| 9 | Patient 12073 | M | 66 | No | “The nurse explained well, it is just that maybe I am stupid or something, because when I got home, I did not know how to use it.” | “Me explicó bien, sólo quizás soy bruto o algo porque cuando llegué a casa no sabía manejarlo.” |
| 10 | Nurse 306 | F | 38 | Yes | “At the beginning, I found it tedious, because we did not have protected hours for this task. I took on more [tasks], because I had more free time. [...] When the [chief] doctor saw that we were having trouble, she said that when we were in the clinic, we should ask the auxiliary nurse to cover for us for a moment [to do our tasks related to CONEMO].” | “Al principio me pareció tedioso porque no teníamos programación. Yo asumía más, porque tenía más tiempo libre. [...] Cuando la doctora vio que estábamos con problemas nos dijeron que si estábamos en el tópico teníamos que decirle al técnico que nos cubra un ratito.” |
| 11 | Nurse 304 | F | 33 | Yes | “[I learned] about 80% [of the procedures] because it was difficult to stay focused during the training, due to the number of tasks that we had [to do] in the healthcare center.” | “[Aprendí] a un 80% porque era difícil concentrarse en el entrenamiento, por las tareas que teníamos en el centro.” |
| 12 | Nurse 304 | F | 33 | Yes | “You, [the research team], were not the problem, but the work burden we have here. We just could not do it, it was pretty uncomfortable because we did not have the time to receive you as we should have. [...] It felt it like ‘another thing’ that I have to do’”. | “La dificultad no eran ustedes [el equipo de investigación], sino la carga laboral que tenemos. No podíamos, era bastante incómodo porque no teníamos el tiempo de atenderlas como debería ser. [...] Lo sentía como ‘otra cosa más que tengo que hacer.” |
| 13 | Nurse 301 | F | 38 | No | “You would have to separate [CONEMO] as an independent program because here each nurse is responsible for one program. It cannot interfere with other activities.” | “Habría que separarlo como un solo programa aparte, porque acá manejamos programas y cada una se dedica a uno. No puede interrumpir las otras actividades.” |
